# Identifying potential novel drugs against *Helicobacter pylori* by targeting the essential response regulator HsrA

**DOI:** 10.1038/s41598-019-47746-9

**Published:** 2019-08-05

**Authors:** Andrés González, Sandra Salillas, Adrián Velázquez-Campoy, Vladimir Espinosa Angarica, María F. Fillat, Javier Sancho, Ángel Lanas

**Affiliations:** 1 Aragon Institute for Health Research (IIS Aragón), San Juan Bosco 13, 50009 Zaragoza, Spain; 2 Institute for Biocomputation and Physics of Complex Systems, Mariano Esquillor (Edif. I + D), 50018 Zaragoza, Spain; 3 Department of Biochemistry & Molecular and Cell Biology, University of Zaragoza, Pedro Cerbuna 12, 50009 Zaragoza, Spain; 4 CIBERehd, Monforte de Lemos 3-5, 28029 Madrid, Spain; 5 ARAID Foundation, Ranillas 1-D, 500018 Zaragoza, Spain; 6 Cancer Science Institute, National University of Singapore, 14 Medical Drive, #12-01, 117599 Singapore, Singapore; 7 Department of Medicine, Psychiatry and Dermatology, University of Zaragoza, Pedro Cerbuna 12, 50009 Zaragoza, Spain; 8 Digestive Diseases Service, University Clinic Hospital Lozano Blesa, San Juan Bosco 15, 50009 Zaragoza, Spain

**Keywords:** Screening, High-throughput screening

## Abstract

The increasing antibiotic resistance evolved by *Helicobacter pylori* has alarmingly reduced the eradication rates of first-line therapies. To overcome the current circulating resistome, we selected a novel potential therapeutic target in order to identify new candidate drugs for treating *H*. *pylori* infection. We screened 1120 FDA-approved drugs for molecules that bind to the essential response regulator HsrA and potentially inhibit its biological function. Seven natural flavonoids were identified as HsrA binders. All of these compounds noticeably inhibited the *in vitro* DNA binding activity of HsrA, but only four of them, apigenin, chrysin, kaempferol and hesperetin, exhibited high bactericidal activities against *H*. *pylori*. Chrysin showed the most potent bactericidal activity and the most synergistic effect in combination with clarithromycin or metronidazole. Flavonoid binding to HsrA occurs preferably at its C-terminal effector domain, interacting with amino acid residues specifically involved in forming the helix-turn-helix DNA binding motif. Our results validate the use of HsrA as a novel and effective therapeutic target in *H*. *pylori* infection and provide molecular evidence of a novel antibacterial mechanism of some natural flavonoids against *H*. *pylori*. The results further support the valuable potential of natural flavonoids as candidate drugs for novel antibacterial strategies.

## Introduction

*Helicobacter pylori* is considered the most prevalent human pathogen worldwide, more than half the world’s population is infected by this Gram-negative bacterium^1^. Unless treated, colonization of the human stomach usually persists lifelong causing gastric inflammation and contributing to the pathogenesis of peptic ulceration, gastric adenocarcinoma, and mucosa-associated lymphoid-tissue (MALT) lymphoma^2,3^. Nearly 90% of all gastric cancers, the third most common cause of cancer death worldwide, can be attributable to *H*. *pylori* infection^1^.

A standard triple therapy containing a proton-pump inhibitor (PPI) and two antibiotics, clarithromycin and either amoxicillin or metronidazole, has been traditionally considered the first-line regimen for treatment of *H*. *pylori* infection^4,5^. However, the PPI-based triple therapy has been described to be losing its efficacy for *H*. *pylori*, with eradication cure rates as low as 50% to 70%, due to high rates of antibiotic resistance, high rates of antibiotic-associated side effects and low compliance^6,7^. Because of increasing failure of the traditional therapy, the current guidelines recommend a quadruple therapy (PPI + amoxicillin + metronidazole + clarithromycin) as first-line strategy, while the PPI triple therapy is restricted to areas with known low clarithromycin resistance^8^. In February 2017, the WHO included *H*. *pylori* in its first ever list of antibiotic-resistant “priority pathogens”, a catalogue of 12 families of bacteria that pose at present the greatest threat to human health^9^. Nowadays, an effective novel therapy against this clinically relevant pathogen is mandatory to increase eradication rates and minimize both antimicrobial resistance and side effects on normal microbiota. To mitigate the challenges of antimicrobial resistance, the search for new bioactive compounds able to inhibit the growth of pathogens by acting specifically on new molecular targets can be a valuable route for drug discovery.

The *H*. *pylori* genome harbours the *hp1043 (hsrA*) gene, encoding for an OmpR-like “orphan” response regulator which has been shown to be essential for cell viability^10,11^. HsrA (for *homeostatic stress regulator*) is unique and highly conserved among members of the Epsilonproteobacteria^12^. The protein functions as a global homeostatic regulator synchronizing metabolic functions and virulence with availability of nutrients and cell division^13^, mediating also the response to oxidative stress^14^. HsrA binds to DNA acting as a transcriptional activator, modulating its own expression^15^, but also the expression of a plethora of genes involved in crucial cellular functions such as translation, transcription, energy metabolism, nitrogen metabolism and redox homeostasis^14,16^. NMR-spectroscopy and X-ray crystallography analyses proposed that HsrA exists as a symmetric dimer *in vivo* with two well-defined functional domains, an N-terminal regulatory domain and a C-terminal DNA-binding/effector domain^17^. Unlike most response regulators, where phosphorylation of the regulatory domain triggers conformational changes that promote dimerization of the protein and activate its DNA binding function, the two domains of HsrA seems to act independently. Thus, mutations in the N-terminal regulatory domain that inhibited dimerization did not affect DNA binding function^17^, while deletion of the C-terminal effector domain did not have effect on the dimerization ability of the protein^15^. Attempts to both deletion and overexpression of HsrA have resulted unsuccessful, supporting not only an essential function of the regulator but also a very tight post-transcriptional control of its expression that ensures appropriate levels of the protein into the cell^12,15^.

As an essential protein for microbial viability, easily produced under lab conditions, with a known crystal structure and no counterpart in humans, the response regulator HsrA become in a promising therapeutic target against *H*. *pylori* infection. In the present study, we screened the Prestwick Chemical Library^®^, a collection of 1120 FDA-approved, off-patent small molecules for compounds that specifically bind to HsrA and potentially inhibit its essential function. Four HsrA inhibitors, the natural flavonoids apigenin, chrysin, kaempferol and hesperetin exhibited strong bactericidal activities against both metronidazole- and clarithromycin-resistant *H*. *pylori* strains.

## Materials and Methods

### Chemicals

The Prestwick Chemical Library was purchased from Prestwick Chemical (France). All drugs were provided as 10 mM solutions in 100% DMSO in 96-well plates, which were stored at −20 °C until use. For some assays, compounds of interest were purchased from Sigma-Aldrich and properly stored according to the manufacturer indications. Stock solutions of each compound were prepared at 20 mM in 100% DMSO for EMSA and ITC, and at 10.24 g/L in 100% DMSO for MIC/MBC determinations. In all cases, stock solutions of drugs were stored at −20 °C.

### Recombinant HsrA expression and purification

The complete sequence of gene *hsrA* (*hp1043*) was amplified from the genomic DNA of *H*. *pylori* strain 26695 (ATCC 700392) using the oligonucleotides Hpylori.hsrA_up (5′- GGAATTCCATATGCGCGTTCTACTGATTG-3′) and Hpylori.hsrA_dw (5′-CCCAAGCTTTTACTCTTCACACGCCGG-3′). The resulting PCR product was digested with *Nde*I and *Hind*III, and inserted between the same restriction sites of vector pET-28a (Novagen). The final vector was partially sequenced to ensure that no modifications in the nucleotide sequence occurred during PCR and cloning. N*-*terminal 6 × His*-*tagged HsrA was overexpressed in *E*. *coli* BL21(DE3) (EMD Biosciences). Recombinant *E*. *coli* carrying the pET-28a-*hsrA* vector was grown to late log phase (OD_600_ = 0.6–0.8) in Luria Bertani (LB) broth at 37 °C with vigorous shaking. At this time, *hsrA* expression was induced by addition of 1 mM IPTG (isopropyl-β-D-thiogalactopyranoside) and culture was further incubated by 6 h. Cells were harvested by centrifugation at 8,000 rpm for 10 min at 4 °C, washed twice with ice-cold PBS (pH 7.4) and stored at −20 °C.

For protein purification, cells were resuspended in ice-cold lysis buffer containing 50 mM Tris-HCl (pH 8), 500 mM NaCl, 10 mM imidazole, 1 mM PMSF (phenylmethylsulfonyl fluoride*)* and sonicated in an ice-water bath by ten 30 s bursts with 30 s cooling intervals. The resulting crude extracts were centrifuged twice at 15,000 rpm for 20 min at 4 °C to remove cell debris. Recombinant His-tagged HsrA was purified by immobilized metal-affinity chromatography (IMAC) using nickel charged Chelating Sepharose Fast Flow resin (GE Healthcare). The bound protein was eluted by using an imidazole gradient (10 mM to 1 M) and dialyzed against the store buffer (50 mM Tris-HCl [pH 8], 300 mM NaCl, 10% glycerol). Protein concentration was determined using the BCA™ Protein Assay kit (Thermo Fisher Scientific). The N-terminal His*-*tag was removed by overnight treatment of the 6 × His*-*tagged HsrA with thrombin (GE Healthcare), 10 units of enzyme for each mg of tagged protein. The cleaved HsrA protein was obtained in the flow throw of a second IMAC-Ni^2+^ purification step.

### High-throughput screening

A target-based high-throughput screening (HTS) methodology was used to test the Prestwich Chemical Library for compounds that specifically bind to *H*. *pylori* HsrA regulator and potentially inhibit its biological function. Compound binding to HsrA was assessed by a fluorescence-based thermal shift assay^18,19^. The screening was performed in V-shape 96-well plates Thermo-Fast 96 (ABgene), adding a final volume of 100 μL of reaction mixture in each well of the plate. A solution of 10 µM of recombinant HsrA was freshly prepared in a buffer containing 50 mM Tris-HCl [pH 8], 150 mM NaCl, 10% glycerol and 5 mM DTT. The SYPRO^®^ Orange ready-to-use fluorescent stain *(*Thermo Fisher Scientific) was added to this protein solution at a final concentration of 10X and thoroughly mixed. Next, 97.5 μL of this reaction mixture was dispensed into all the wells of a V-shape 96-well plate. While all wells of the columns 1 and 12 received 2.5 μL of DMSO (vehicle) instead of compounds and were used as reference controls, each of the eighty wells of columns 2 to 11 in the 96-well plate received 2.5 μL of a different compound from the chemical library (250 μM as final concentration each). After mixing, each well of the plate was overlaid with 50 μL of mineral oil to avoid evaporation. Unfolding curves were registered from 25 °C to 75 °C in 1 °C steps using a FluoDia T70 High Temperature Fluorescence Microplate Reader (Photon Technology International, UK). The system was allowed to equilibrate at each temperature for 1 min before each fluorescence acquisition. In practice, this represents an operational heating rate of 0.25 °C/min approximately. Because SYPRO Orange dye interacts with hydrophobic regions in the protein that become exposed upon denaturation, fluorescence emission of the dye serves as a read out of thermal denaturation of the protein^20^. The unfolding curves corresponding to each well were analysed using a homemade software that estimates the midpoint temperature of unfolding (*T*_m_) of each well. Compounds that increased thermal stability of HsrA above the twofold standard deviation value of reference controls were identified as HsrA binders and selected as potential inhibitors.

### Electrophoretic mobility shift assays

The DNA binding activity of the recombinant HsrA response regulator as well as its putative inhibition by HsrA binders were evaluated by electrophoretic mobility shift assays (EMSAs). A 300-bp promoter region of *porGDAB* operon was amplified by PCR and used as target sequence of HsrA in all EMSA experiments^14^. Recombinant HsrA protein (6 μM) was mixed with 120 ng of target promoter in a 20 μL reaction volume containing 10 mM bis-Tris (pH 7.5), 40 mM KCl, 100 mg/L BSA, 1 mM DTT, and 5% glycerol. During the inhibition assays, putative inhibitors were added to final concentrations of 2, 1, 0.5 and 0.1 mM to the mixtures of protein and DNA. Binding assays with DMSO instead of inhibitors were included as vehicle controls. To ensure the specificity of EMSAs, a 150-bp DNA fragment corresponding to the coding region of gene *pkn22* (*alr2502*) from *Anabaena* sp. PCC 7120 was included as non-specific competitor DNA in all assays. Mixtures were incubated at room temperature for 20 min and subsequently separated on a 6% non-denaturing polyacrylamide gel in running buffer (25 mM Tris, 190 mM glycine) at 90 V. The gel was stained with SYBR Safe DNA gel stain (Invitrogen) and processed with a Gel Doc 2000 Image Analyzer (Bio-Rad).

### Minimal inhibitory and bactericidal concentrations

The antibacterial activity of HsrA inhibitors was tested against three different strains of *H*. *pylori*: ATCC 700392, ATCC 43504 (metronidazole-resistant reference strain), and ATCC 700684 (clarithromycin-resistant reference strain). MIC determinations were carried out by the broth microdilution method as previously described^18,21^, with slight modifications. *H*. *pylori* strains were grown in Blood Agar Base N°2 (OXOID) supplemented with 8% defibrinated horse blood (OXOID) in a humidified microaerobic incubator (85% N_2_, 10% CO_2_, 5% O_2_) at 37 °C for 72 h. To prepare inoculums for MIC assays, bacteria were grown for 48 hours at 37 °C in brain heart infusion broth supplemented with 4% FBS (BHI + FBS) and next diluted in the same fresh medium to a final optical density at 660 nm of 0.01 (∼10^6^ CFU/mL). From this inoculum, 195 µL were added to all wells of the first column of a sterile 96-well flat-bottom microtiter plate, while 100 µL were added to all wells from column 2 to 12. Next, 5 µL of a 10.24 g/L in 100% DMSO stock solution of each HsrA inhibitor were added to wells of the first column and two-fold serial dilutions were performed in order to test each inhibitor in a concentration range from 256 to 0.125 mg/L. DMSO, metronidazole and clarithromycin were included as controls in all assays. Plates were incubated under microaerobic conditions at 37 °C and examined visually after 72 h^22,23^. MIC values were defined as the lowest concentration of compound that completely inhibited the visible growth of bacteria after 72 h incubation. For MBC determinations, 10 μL aliquots of two compound dilutions around the MIC value were seeded on Blood Agar Base N°2 supplemented with 8% defibrinated horse blood and incubated for 72 h under microaerobic conditions at 37 °C. MBC was defined as the lowest concentration of compound that prevented the growth of ≥99.9% of *H*. *pylori* cells after subculture on the inhibitor-free medium. Each experiment was performed twice in triplicate to confirm the results.

### Time-kill kinetics assays

Time-kill kinetics of selected flavonoids were carried out as previously described^18^ with slight modifications. A final concentration of 2 × MIC from each flavonoid was mixed with 200 µL of *H*. *pylori* strain ATCC 700684 at 1.0 × 10^5^ CFU/mL in BHI + FBS. The experiments were performed twice in triplicate using sterile 96-well flat-bottom microtiter plates. Plates were incubated under microaerobic conditions at 37 °C with shaking. Mixtures of bacteria with the same amount of DMSO (vehicle) instead flavonoid were incubated under the same conditions and included as controls in all determinations. Aliquots of 20 µL taken at time intervals of 0, 2, 4, 8, and 24 h were serially diluted in BHI medium and plated in Blood Agar Base N°2 supplemented with 8% defibrinated horse blood nutrient agar for colony forming unit (CFU) determination. Plates were incubated at 37 °C for 72 h under microaerobic conditions. The amount of grown *H*. *pylori* colonies was counted and presented as log_10_ CFU/mL versus incubation time. The results were subjected to statistical analysis using the Mann-Whitney *U* test^24^. The criterion *P* < 0.1 was used to determine the statistical significance.

### Checkerboard assay

The existence of synergism in the antimicrobial activity of selected flavonoids and the first-line antibiotics clarithromycin and metronidazole was determined against two *H*. *pylori* strains (clarithromycin-resistance strain ATCC 700684 and metronidazole-resistant strain ATCC 43504) using the checkerboard assay^25,26^.

Solutions at 4-fold the highest concentration to be tested were prepared for each compound. Then, antibiotic and flavonoid were two-fold serially diluted in BHI + FBS using two sterile 96-well flat-bottom microtiter plates; one of the compounds was diluted along the rows (horizontally) of a first plate, and the other compound was diluted along the columns (vertically) of the second plate. A matrix of both gradient was obtained by mixing 50 µL of the content of each well of plate 1 (except from first column) with 50 µL from the same well of plate 2 (except from last row), thereby diluting both compounds at two-fold the concentrations to be tested. Thus, the first column in plate 3 would have serial dilutions of just one of the compounds, and the last row would have serial dilutions of the other. Finally, 100 µL of freshly prepared inoculum (2×10^6^ CFU/mL) of proper *H*. *pylori* strain were added to all wells. The plates were incubated for 72 h at 37 °C under microaerophilic conditions. Then, 30 µL of 0.1 mg/ml filter-sterilized resazurin (Sigma-Aldrich) was added as growth indicator^25,27^ to each well and plates were further incubated for 8 hours. Each tested panel was performed in duplicate. The interaction between the tested antimicrobial agents was determined by calculating the fractional inhibitory concentration (FIC) index. In the 96-well plate, the first column shows the MIC of one of the compounds (A), and the last row shows the MIC of the second compound (B). FIC index could be calculated as: FIC_A_ (MIC_A in the presence of B_/MIC_A alone_) + FIC_B_ (MIC_B in the presence of A_/MIC_B alone_). The FIC index allows objective identification of any interaction between two compounds against a particular bacterial strain, indicating whether there is synergy (FIC index ≤0.5), additive effect (FIC index >0.5 to ≤1), no interaction or neutral (FIC index >1 to ≤4), or antagonism (FIC index >4)^26,28,29^. The term “synergy” implies that the resulting effect of a combination is significantly greater than the sum of its individual parts, while “additive effect” occurs when substances added together will improve or increase their efficacies, albeit not to the extent of a synergistic interaction^30^.

### Isothermal titration calorimetry assays

Direct interaction of the selected compounds with HsrA was assessed by isothermal titration calorimetry (ITC). Titrations were performed in a high-sensitivity AutoiTC200 microcalorimeter (MicroCal, Malvern Instruments). Protein samples and compound solutions were properly degassed and carefully loaded into the calorimetric cells to avoid bubble formation during stirring. Experiments were performed with freshly prepared buffer-exchanged protein solutions, at 25 °C in 50 mM Tris-HCl [pH 8], 150 mM NaCl, 10% glycerol, 1% DMSO. A solution of HsrA (20 µM) in the calorimetric cell was titrated with a solution of the corresponding compound (200 µM) located in the injecting syringe. A sequence of 19 injections of 2 µL volume was programmed with a time spacing of 150 s, a stirring speed of 750 rpm, and a reference power of 10 µcal/s in the sample cell^31,32^. The binding isotherm (ligand-normalized heats as a function of the molar ratio) was obtained through integration of the individual heat effect associated with each ligand injection observed in the thermogram (thermal power required to maintain the difference between the temperatures of the sample cell and the reference cell close to zero as a function of time). Dissociation constants and binding enthalpies were calculated by non-linear least squares regression data analysis in Origin 7.0 (OriginLab).

### Molecular docking

The tridimensional structures of flavonoids (ligands) were downloaded from PubChem^33^ and were docked onto the structure of the *H*. *pylori* HsrA response regulator (2HQR, model 1, chain A) obtained from the PDB^34^, using AutoDock Vina^35^. The ligands were considered flexible, and a ligand-specific torsion tree was defined in each case, representing the rigid and rotatable pieces of the molecule. In each torsion tree rotatable bonds were allowed the torsional degrees of freedom corresponding to the specific bond, according to AutoDock torsion parameterizations. The structure of the protein was considered rigid and a grid enclosing the whole structure of the protein was defined, representing the search space for putative interaction sites. Separately, pre-calculated maps were defined for the ligands, encompassing independent maps for each atom type in the ligand, combining the desolvation and electrostatics potentials. The AutoGrid algorithm was then used to estimate the interaction energy of a ligand conformation (i.e. ligand pose) by summing up the contribution of atoms of a specific element at each point in the grid around the rigid receptor. After scanning the whole protein structure search space, the docking solutions passing a threshold of ΔG < −4.0 kcal/mol were ranked based on the estimated interaction energy. The top ranking pose for each ligand was selected as predicted model of interaction.

## Results

### High-throughput screening of Prestwick Chemical Library reveals specific flavonoid binders of *H. pylori* HsrA response regulator

**Figure 1:**
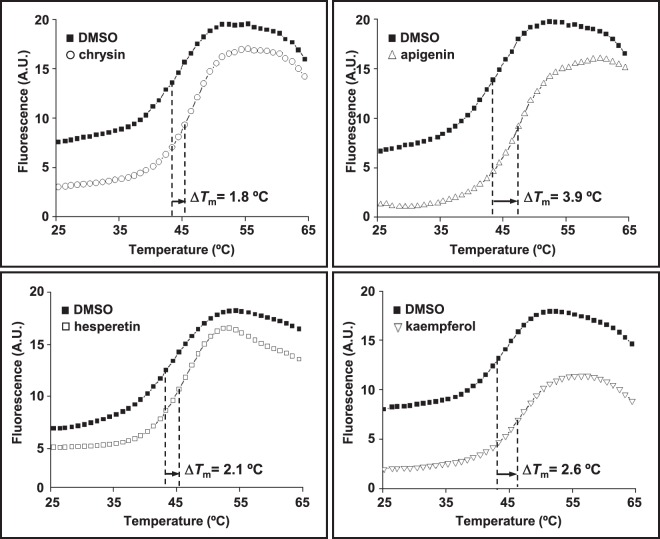
Thermal upshifts of the HsrA unfolding curve triggered by 250 μM of the natural flavonoids chrysin, apigenin, hesperetin and kaempferol. The HTS was carried out using a fluorescence-based thermal shift assay. Any compound that preferentially binds to the native state of HsrA would increase the protein stability and cause an upshift of the protein unfolding curve due to the increased melting temperature (*T*_m_) of the protein-binder complex. In all assays, DMSO (vehicle) instead of compounds were mixed with the protein and used as reference control of the protein unfolding curve.

As the first step to identify new candidate drugs for treating *H*. *pylori* infection, we screened the Prestwich Chemical Library for small FDA-approved molecules that specifically bind to the essential response regulator HsrA. The HTS was carried out using a fluorescence-based thermal shift assay^18,19^. With this method, any compound of the chemical library that preferentially binds to the native state of HsrA increases the protein conformational stability and causes a shift of the protein unfolding curve to higher temperatures due to the increased melting temperature (*T*_m_) of the protein-binder complex. To minimize the identification of false-positive binders, we defined as relevant compounds those which increased the thermal stability of HsrA above the twofold standard deviation of *T*_m_ value of reference controls. Following this selection criterion, the target-based HTS approach identified 14 compounds of the 1120 compound collection (1.25%) as HsrA binders. Notably, seven of these positive hits consisted in naturally occurring flavonoids, including apigenin, chrysin, hesperetin, kaempferol, luteolin, myricetin, and quercetin (Table 1). The increases in *T*_m_ values indicated that all of these seven natural flavonoids bound to HsrA and formed stable complexes. The thermal upshifts of the HsrA unfolding curve triggered by 250 μM of four different flavonoids (chrysin, apigenin, hesperetin and kaempferol) are shown in Fig. 1. Due to the high heterogeneity of the rest of HsrA binders (data not shown), we focused our further analyses to primarily discern the potentiality of flavonoid binders of HsrA as phytochemical therapies to *H*. *pylori* infections.

**Table 1:** HsrA binders identified by a fluorescence-based HTS method using the thermal shift assay.

Compound	Δ*T*_m_ (°C)^a^	Class	Chemical structure	Pharmacological effects
chrysin	1.8	flavone		anti-inflammatory, antineoplastic, antioxidant, hepatoprotector
apigenin	3.9	flavone		antiproliferative, anti-inflammatory
luteolin	1.8	flavone		anti-inflammatory, antioxidant, antiproliferative
hesperetin	2.1	flavanone		cholesterol lowering, hypolipidemic, antioxidant, anti-allergic, antineoplastic, anti-inflammatory
kaempferol	2.6	flavonol		anti-inflammatory, diuretic, antioxidant
quercetin	4.2	flavonol		antioxidant, anti-allergic, anti-inflammatory, antiproliferative
myricetin	3.9	flavonol		antineoplastic, antioxidant, hypoglycemic

^a^Increase in the *T*_m_ value of protein-ligand complex with respect to the mean *T*_m_ value of controls (protein + DMSO). In all cases, Δ*T*_m_ > twofold standard deviation of controls.

### Flavonoids inhibit DNA binding activity of HsrA *in vitro*

**Figure 2:**
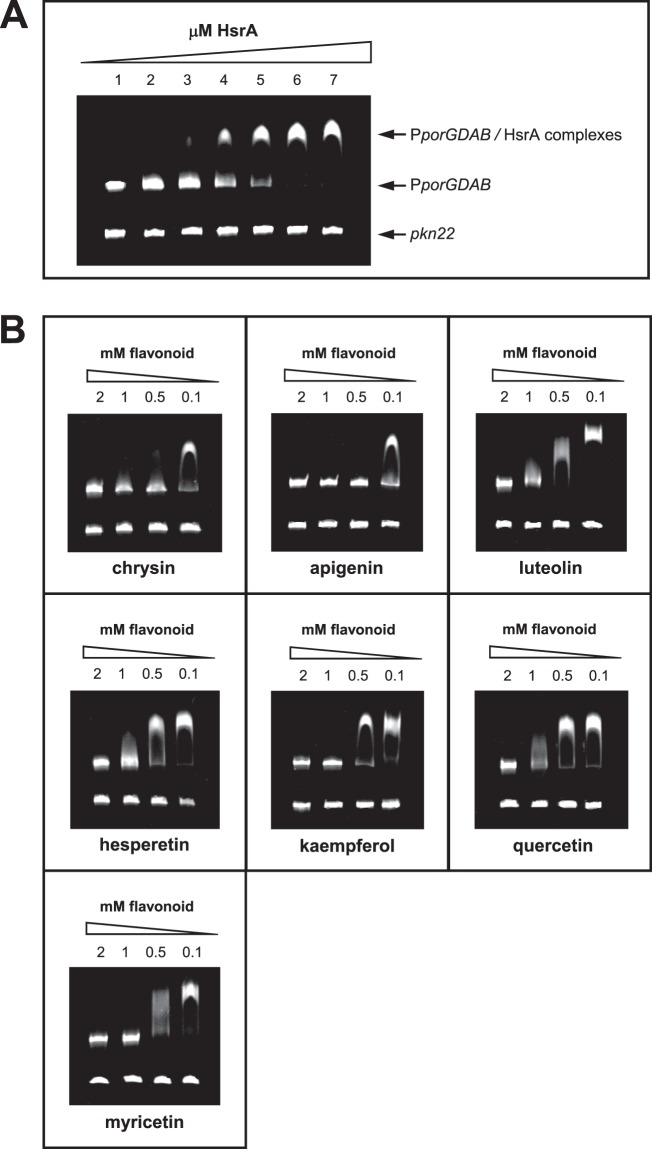
Impact of several natural flavonoids on the *in vitro* affinity of HsrA for its target promoter. **(A)** EMSAs showing the ability of HsrA to specifically bind *in vitro* the promoter region of *porGDAB* operon. DNA fragments were mixed with increasing concentrations of recombinant HsrA protein (indicated in μM) and separated on a 6% PAGE. A 150-bp DNA fragment of *Anabaena* gene *pkn22* was included as non-specific competitor DNA in all assays. **(B)** DNA fragments were mixed with 6 μM of recombinant HsrA protein in the presence of 2, 1, 0.5 and 0.1 mM of natural flavonoids.

HsrA is an orphan response regulator that functions as a transcriptional activator of gene expression; hence, it interacts with DNA and binds to specific sequences located in target promoters. Biological activity of purified recombinant HsrA was evaluated before its use in the target-based HTS in order to ensure that the native conformation of the response regulator interacts with potential inhibitors of the chemical library. EMSA analyses of the recombinant response regulator in the presence of its target promoter P_*porGDAB*_^14^ demonstrated a high affinity of HsrA by its target DNA in a concentration-dependent manner (Fig. 2A). Under the experimental conditions used in EMSAs, 120 ng of target DNA were completely and specifically complexed to HsrA from 6 μM of the recombinant protein. Hence, this concentration was subsequently used for EMSA inhibition assays in the presence of flavonoid binders.

To determine whether the flavonoid binders previously identified with our screening approach effectively inhibit the DNA binding activity of HsrA, we carried out EMSA analyses in the presence of different concentration of each flavonoids (from 2 mM to 100 μM). Since flavonoids were diluted in 100% DMSO, negative controls of EMSAs were carried out by mixing the same amounts of protein and DNA in the presence of DMSO instead of flavonoids. As shown in Fig. 2B, all flavonoids appreciably inhibited the *in vitro* binding activity of HsrA to its target promoter. However, the *in vitro* inhibitory effects of some flavonoid binders such as apigenin or chrysin on the DNA binding activity of HsrA appeared noticeable stronger than those exerted by other flavonoids such as quercetin.

### Apigenin, chrysin, kaempferol and hesperetin exhibit potent bactericidal activity against *H. pylori*

**Figure 3:**
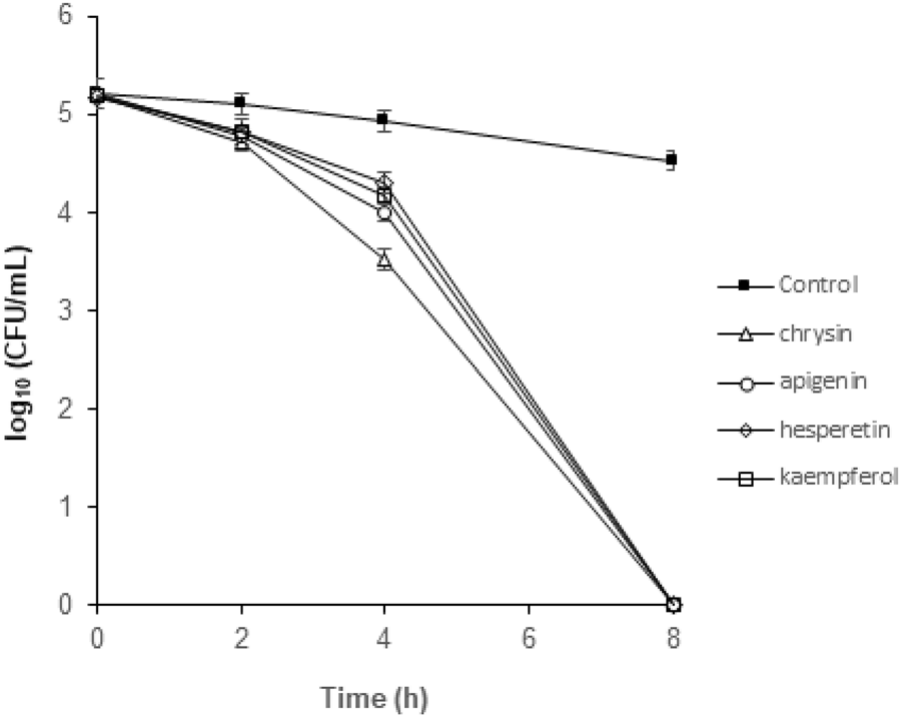
Time-kill kinetics of natural flavonoids chrysin, apigenin, hesperetin and kaempferol against *H*. *pylori* strain ATCC 700684. Bacterial counts were determined at time zero and after 2, 4, 8, and 24 hours of incubation with two times the MIC. Mixtures of bacteria with DMSO (vehicle) instead flavonoid were used as controls. Values are the averages of three independent determinations; vertical bars represent standard deviations. Please note that in some instances the error is smaller than the symbols used.

To evaluate the antibacterial activity of the HsrA inhibitors, we determined both MIC and MBC values of each HsrA inhibitors against three different strains of *H*. *pylori*, including the metronidazole-resistant strain ATCC 43504 and the clarithromycin-resistant strain ATCC 700684. In all cases, the lowest concentration of compound that completely inhibited the visible growth of bacteria after 72 h incubation was also the same which prevented the growth of ≥99.9% of *H*. *pylori* cells after subculture on the inhibitor-free medium (Table 2). Thus, both MIC and MBC values corresponded to the same concentration of compound for all the HsrA inhibitors tested. Despite all the seven natural flavonoids specifically bound to HsrA and inhibited its biological function *in vitro* according to EMSA, only four of them, apigenin, chrysin, kaempferol and hesperetin, exhibited potent bactericidal activities (MBC ≤ 8 mg/L) against *H*. *pylori*. Hence, the bactericidal effect of the other three HsrA inhibitors, luteolin, quercetin, and myricetin were evidenced at concentrations quite higher than MIC breakpoints for antimicrobials traditionally used in anti-*H*. *pylori* therapies^36,37^, thereby these three flavonoids were considered poorly active against *H*. *pylori*. To further evaluate the bactericidal activities of apigenin, chrysin, kaempferol and hesperetin against *H*. *pylori*, time-kill kinetics were carried out at 2 × the MIC values (listed in Table 2) for each compound against the *H*. *pylori* strain ATCC 700684. As in shown Fig. 3, no live bacteria could be detected at 8 hours after exposition to 2 × MIC of each flavonoid, and this was found to be statistically significant according to the Mann-Whitney *U* test^24^ (*P* < 0.1). The bactericidal activity of chrysin at this concentration was significant (*P* < 0.1) even after only 4 hours of exposition to the microorganism.

**Table 2:** Minimal inhibitory and bactericidal concentrations of HrsA inhibitors.

Compound	MIC (MBC), mg/L		
	ATCC 700392	ATCC 43504 (MTZ-R)	ATCC 700684 (CLR-R)
chrysin	8 (8)	8 (8)	4 (4)
apigenin	8 (8)	8 (8)	8 (8)
luteolin	64 (64)	32 (32)	64 (64)
hesperetin	8 (8)	8 (8)	4 (4)
kaempferol	8 (8)	16 (16)	16 (16)
quercetin	64 (64)	64 (64)	64 (64)
myricetin	128 (128)	128 (128)	128 (128)
metronidazole	1 (2)	64 (128)	1 (2)
clarithromycin	≤0.12 (≤0.12)	≤0.12 (≤0.12)	8 (16)

MTZ-R, metronidazole resistant strain. CLR-R, clarithromycin resistant strain.

The *in vitro* interactions of the two flavonoids that exhibited the most potent bactericidal activities, chrysin and hesperetin (Table 2), with antibiotics commonly used as first-line *H*. *pylori* eradication therapies were analysed. As shown in Table 3, the combinations of flavonoids with antibiotics inhibited the growth of *H*. *pylori* at a lower concentration than when each single compound was assayed separately. In all cases, the combination of chrysin or hesperetin with clarithromycin or metronidazole showed a synergistic or additive antimicrobial effect against the two antibiotic-resistant strains used in the assays. Notably, chrysin reduced the MIC value of clarithromycin up to 8 times (FIC = 0.125) in the clarithromycin-resistant strain ATCC 700684, and up to 16 times the MIC value of metronidazole (FIC = 0.0625) in the metronidazole-resistant strain ATCC 43504 (Table 3).

**Table 3:** Interaction of the natural flavonoids chrysin and hesperetin with antibiotics of first-line *Helicobacter pylori* eradication therapies in two *H*. *pylori* resistant strains.

Strain	Combination tested	FIC_antibiotic_	FIC_flavonoid_	FICI^a^	Interaction^b^
ATCC 700684 (CLR-R)	CLR + chrysin	0.125	0.25	0.375	synergy
	CLR + hesperetin	0.5	0.5	1	additive
ATCC 43504 (MTZ-R)	MTZ + chrysin	0.0625	0.0625	0.125	synergy
	MTZ + hesperetin	0.25	0.25	0.5	synergy

^a^Fractional inhibitory concentration (FIC) index could be calculated as: FIC_A_ (MIC_A in the presence of B_/MIC_A alone_) + FIC_B_ (MIC_B in the presence of A_/MIC_B alone_).

^b^According to the FICI value, the interaction between two compounds against a particular bacterial strain can be classified as: synergy (FICI ≤ 0.5), additive (FICI > 0.5 to ≤1), no interaction or neutral (FICI > 1 to ≤4), and antagonism (FICI > 4).

### Bactericidal flavonoids interact with the C-terminal effector domain of HsrA

**Figure 4:**
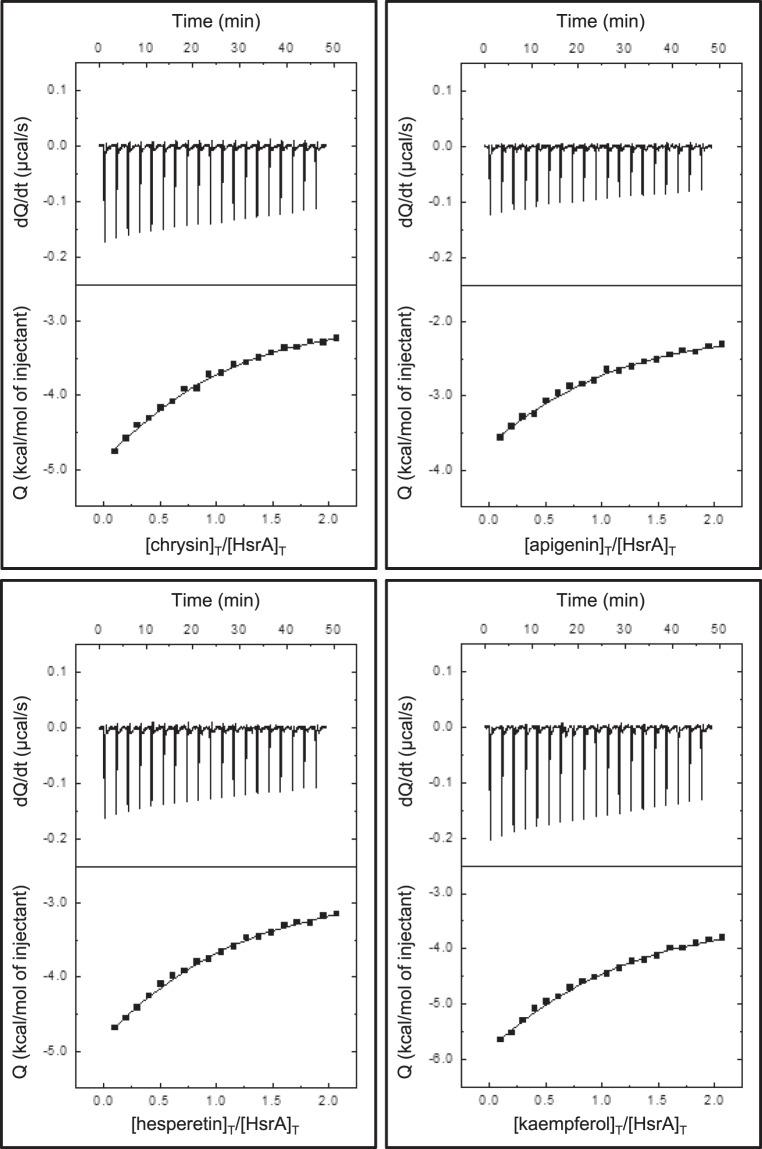
Isothermal titration calorimetry analyses of the *H*. *pylori* HsrA response regulator interactions with the natural flavonoids chrysin, apigenin, hesperetin and kaempferol. In the figures, upper panels show the ITC thermograms while lower panels show the binding isotherms.

ITC and molecular docking analyses were carried out in order to analyze the binding affinity of bactericidal flavonoids (apigenin, chrysin, kaempferol and hesperetin) for the target protein as well as to understand the structural basis of this interaction. The ITC data indicated dissociation constants in the micromolar range in all cases, while complexes of a 1:1 stoichiometry were observed for all flavonoids (Table 4, Fig. 4). Thus, each HsrA monomer appeared to bind one molecule of flavonoid.

**Table 4:** Analyses of the interaction between HsrA and its bactericidal inhibitors by isothermal titration calorimetry and molecular docking.

Compound	ITC^a^				Molecular Docking^b^
	n	*K*_d_ (µM)	ΔH (kcal/mol)	ΔG (kcal/mol)	Interacting residues
chrysin	0.77	17	−4.1	−6.5	**P181**, **K165**, **I184**
apigenin	0.81	20	−3.8	−6.4	**P198**, I135, **K194**, V144, G146, K145, L152
hesperetin	0.83	26	−5.8	−6.2	**P198**, **M195**, **I191**, **K194**, P148, V144, K145, G146, L152
kaempferol	0.78	33	−8.6	−6.1	Y137, **K194**, P148, G146

^a^Absolute error in n is 0.06, relative error in *K*_d_ is 40%, absolute error in ΔH is 0.4 kcal/mol, absolute error in ΔG is 0.2 kcal/mol.

^b^Amino acid residues directly involved in forming the helix-turn-helix (HTH) DNA binding motif of HsrA are highlighted in bold fonts.

**Figure 5:**
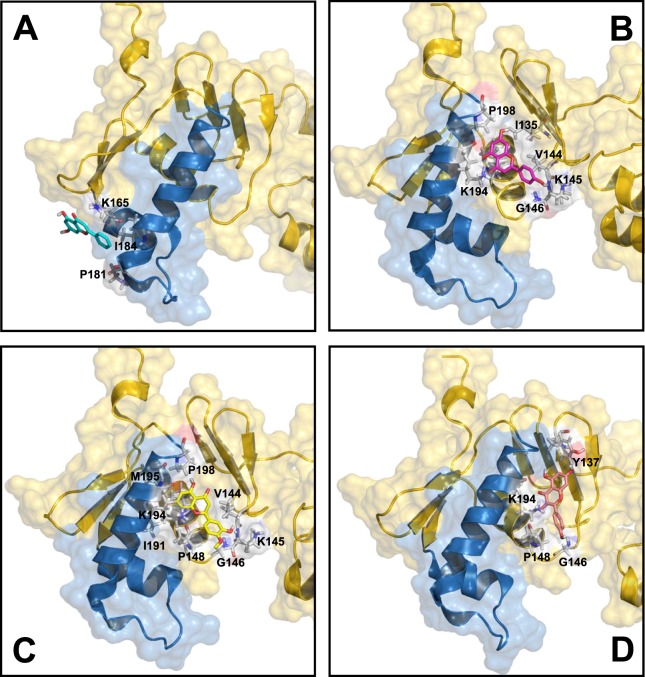
Local overviews of the best ranked docking poses of chrysin **(A)**, apigenin **(B)**, hesperetin **(C)**, and kaempferol **(D)** interaction with HsrA. Ribbon model and transparent molecular surface showing the interacting residues of HsrA to each flavonoid. The helix-turn-helix (HTH) DNA binding motif of HsrA is highlighted in blue. Some interacting residues are indicated.

As shown in Fig. 5, flavonoid binding to the native conformation of HsrA occurs preferably at the C-terminal effector domain, as predicted by molecular docking analyses. Notably, the predicted binding site for chrysin differed from those occupied for the other three flavonoids. However, the best binding poses for all bactericidal flavonoids appeared to interact with amino acid residues specifically involved in forming the helix-turn-helix (HTH) DNA binding motif of the HsrA response regulator (Table 4)^17^.

## Discussion

The dramatic increase in bacterial resistance to antibiotics belonging to all pharmacological classes constitutes a major worldwide health concern and it must be a priority to the scientific community^38^. An important challenge in the current antibiotic resistance crisis is the identification of novel microbial targets, essential for *in vivo* growth or pathogenicity, whose inhibitors can overcome the currently circulating resistome of human pathogens. Among the plethora of genes indispensable for *in vivo* growth and virulence of bacterial pathogens, transcriptional regulators (TRs) stand out because of the potential multitargeting effect of anti-TR molecules on cellular virulence and physiology^39^.

In *H*. *pylori*, the essential response regulator HsrA appears as a promising target for drug development^11–16^. In the present study, we carried out an affinity-based HTS^18,19^ of the Prestwick Chemical Library for FDA-approved molecules that specifically bind to the HsrA response regulator and potentially inhibit its essential biological function. By using this screening methodology, seven natural flavonoids including apigenin, chrysin, hesperetin, kaempferol, luteolin, myricetin, and quercetin were identified as HsrA binders, since all of them significantly increased its thermal stability. All flavonoids identified as HsrA binders appreciably inhibited the DNA binding activity of HsrA *in vitro* according to EMSA; however, only four of them, apigenin, chrysin, kaempferol and hesperetin, exhibited potent bactericidal activities against three different strains of *H*. *pylori*, including strains resistant to metronidazole and clarithromycin. Notably, the flavonoids with two or more hydroxyl groups in ring B (luteolin, quercetin, myricetin) showed moderate to low bactericidal activities against *H*. *pylori*. Since more hydroxyl groups results in lower hydrophobicity^40^, the lowest bactericidal activity of myricetin despite its HsrA inhibitory effect could be the result of poor translocation across the cell membrane. Among all the flavonoids evaluated in this work, the flavone chrysin showed the most potent bactericidal activity and the most synergistic effect in combination with antibiotics of first-line *H*. *pylori* eradication therapies. Notably, chrysin strongly synergized with both clarithromycin and metronidazole, supporting its valuable potential as adjuvant in current combinatory therapies, especially in the treatment of refractory *H*. *pylori* infections or multidrug-resistance isolates^41,42^.

HsrA functions as a symmetric dimer *in vivo* with two well-defined functional domains in each monomer, an N-terminal regulatory domain (residues 1 to 119) and a C-terminal DNA-binding/effector domain (residues 120 to 223)^17^. In contrast to most response regulators, the two domains of HsrA seem to act independently, and mutations in the N-terminal regulatory domain did not appreciably affect DNA binding function^17^. Each monomer of HsrA contains eight α-helices and twelve β-strands, where two α-helices in the C-terminal effector domain (α7 and α8, comprising the residues 165 to 199) form the HTH DNA binding motif. In fact, single mutations at the amino acid residues involved in forming the HTH motif or its periphery noticeably inhibited or completely destroyed the DNA binding ability of the protein^17^. Molecular docking analyses suggested that the bactericidal flavonoids apigenin, chrysin, kaempferol and hesperetin bind to HsrA preferably in its C-terminal effector domain, interacting with several amino acids involved in both the structure of the HTH motif and the domain stabilization. According to these *in silico* predictions, the HsrA activity inhibition by flavonoids could be the result of direct blockage of key amino acid residues involved in protein-DNA interaction and/or conformational changes in the effector domain that destabilize the regulator binding to target promoters. In fact, one of the molecular mechanisms associated to the antibacterial action of flavonoids is the formation of complexes with proteins through different forces such as hydrogen bonding, hydrophobic interactions and covalent bonds, thereby inactivating microbial adhesins, enzymes, transport proteins, and other cell targets^43^.

Flavonoids comprise a wide class of compounds with variable phenolic structures, mostly found in plants^44^. Both medicinal plants and flavonoid rich extracts have been used for centuries to treat human pathologies^45,46^, including *H*. *pylori* infection and related diseases^47–50^. A large number of studies have demonstrated the strong antibacterial activity of natural, semisynthetic^51^ and synthetic flavonoids^52^; however, their mechanisms of action remain poorly characterized. In *H*. *pylori*, flavonoids have been found to inhibit enzymatic activities such as urease^53^, DNA gyrase^48^, dihydrofolate reductase^48^, *N*-acetyltransferase^54^, D-alanine:D-alanine ligase^55^, and β-hydroxyacyl-acyl carrier protein dehydratase^56^. Quercetin inhibited vacuolation and caspase-3 activation in HeLa cells induced by the vacuolating cytotoxin A, protecting from gastric inflammation and apoptosis associated with *H*. *pylori* infection^57,58^. Kaempferol decreased transcription of type IV secretion system (T4SS) components involved in CagA injection and secretion system subunit protein A (SecA) of type V secretion system (T5SS) involved in VacA secretion, thereby producing an anti-inflammatory effect by suppressing the translocation of CagA and VacA proteins^59^. Apigenin decreased atrophic gastritis and gastric cancer progression in Mongolian gerbils^60^, while hesperetin appears to damage the cell membrane triggering bacterial lysis^51^. Notably, the results of different studies conducted to decipher the mechanisms of action of flavonoids as antimicrobial agents suggest that a single flavonoid molecule could have multiple targets in the cell, triggering bacterial death or virulence decrease by inhibiting and/or affecting the activities of several biomolecules at the same time. Thus, apigenin, one of the HsrA inhibitors and potent anti-*H*. *pylori* bactericidal flavonoid identified in this work, also functions as inhibitor of the enzymes D-alanine:D-alanine ligase^55^ and β-hydroxyacyl-acyl carrier protein dehydratase^56^. Similarly, the lipophilic flavonoid hesperetin inhibited HsrA activity but also affected the integrity of *H*. *pylori* cell membrane^51^. In the last years, flavonoids have gained attention not only for its well-documented antimicrobial action, but also because some of these natural compounds manifest ability to reverse the antibiotic resistance in certain pathogens and enhance the action of some current antibiotic drugs^61–63^. Hence, combinatory therapies that take advantage of the clear evidences of synergism between flavonoids and several conventional antibiotics is now considered a promising strategy in the clinical therapy of infections with antibiotic-resistant microorganisms such as methicillin-resistance *Staphylococcus aureus* (MRSA)^64^.

Overall, the results of the present study validate the use of the response regulator HsrA as a novel and effective therapeutic target in *H*. *pylori* infection and further support the great potential of natural flavonoids as pharmaceutical candidates for novel antibacterial or antivirulence chemotherapies. In addition, our results provide molecular evidence of a novel antibacterial mechanism of some natural flavonoids against *H*. *pylori* infection and demonstrate that this mechanism of action efficiently overcome the strategies evolved by this pathogen to evade the effect of first-line eradication therapies such as metronidazole and clarithromycin.

## Data Availability

Materials, data and protocols are presented within the manuscript.
